# Arthroscopic Anterior Cruciate Ligament Repair With Suture Augmentation Provides Good to Excellent Midterm Functional Outcomes Despite Clinically Relevant Revision Rates

**DOI:** 10.1002/ars2.70128

**Published:** 2026-10-23

**Authors:** Anna Patricia Egert, Kristian Nikolaus Schneider, Georg Gosheger, Jan Christoph Theil, Jan Frederic Weller, Alexander Klug, Georg Ahlbäumer

**Affiliations:** ^1^ Department of Orthopaedics and Trauma Surgery BG Unfallklinik Frankfurt am Main Frankfurt am Main Germany; ^2^ Department of Orthopaedics and Tumor Orthopaedics University Hospital of Münster Münster Germany; ^3^ Department of Hematology and Oncology Including Pneumology University Medical Center Hamburg‐Eppendorf (UKE) Hamburg Germany; ^4^ Department of Orthopaedics and Trauma Surgery Klinik Gut St. Moritz Switzerland

## Abstract

**Purpose:**

To evaluate midterm functional outcomes after anterior cruciate ligament (ACL) repair with suture augmentation and to determine whether concomitant injuries influence patient‐reported outcomes.

**Methods:**

This retrospective single‐center cohort study included patients undergoing arthroscopic ACL repair with suture augmentation between January 2017 and March 2019. Eligible patients had proximal ACL tears (Sherman type I‐II) with adequate tissue quality at a minimum 6‐year follow‐up. Patient‐reported outcome measures included the International Knee Documentation Committee, Lysholm score, Tegner activity score, and forgotten joint score. Multivariate regression analysis was performed to identify predictors of postoperative outcomes. Clinically meaningful improvement was assessed using the minimal clinically important difference.

**Results:**

Of 93 patients, 80 (86%) were available for follow‐up. Thirteen patients (16.3%) underwent revision surgery, all consisting of ACL reconstruction, at a median time of 35 months (interquartile range [IQR] 8‐49) and were excluded, leaving 67 patients for evaluation. At a median follow‐up of 79 months (IQR 75‐97), median International Knee Documentation Committee was 94 (IQR 71‐100); Lysholm score, 92 (IQR 74‐100); and forgotten joint score, 86 (IQR 65‐94). Median Tegner activity score decreased from 7 (IQR 4‐10) preinjury to 6 (IQR 4‐9) postoperatively (*P* = .03), remaining within the minimal clinically important difference in 74.6% of patients. Concomitant meniscal (n = 41) and ligamentous injuries (n = 63) were addressed intraoperatively and did not significantly affect patient‐reported outcome measures. Delayed surgery was the only independent predictor of lower postoperative Tegner activity scores (*β* = −0.79, *P* = .025).

**Conclusions:**

Arthroscopic ACL repair with suture augmentation achieved good to excellent midterm functional outcomes in a 6‐year follow‐up, with 74.6% of patients maintaining activity levels within the minimal clinically important difference threshold. However, the revision rate of 16.3% remains clinically relevant. Concomitant ligamentous and meniscal lesions of low severity did not result in inferior patient‐reported outcomes in this cohort.

**Level of Evidence:**

Level IV, retrospective therapeutic case series.

## Graphical abstract

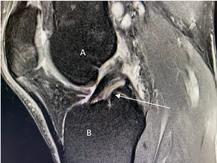

Anterior cruciate ligament (ACL) injuries are among the most frequent knee injuries in physically active individuals and athletes.^1^ In the past, ACL repair was performed but later abandoned because of poor outcomes and high failure rates.^2^ Consequently, ACL reconstruction using autografts or allografts became the gold standard for restoring knee stability and function.^2^ However, reconstruction is associated with donor‐site morbidity, graft failure risk, and prolonged rehabilitation.^3^ Recently, techniques such as arthroscopic ACL repair with suture augmentation (SA) have renewed the interest in primary repair.^4^

It enhances biomechanical stability, supports biological healing, and preserves native proprioceptive fibers. Potential advantages include faster recovery and avoidance of graft harvest complications.^5^, ^6^ Early and midterm studies show promising outcomes with high levels of patient satisfaction and functional improvement.^7^, ^8^, ^9^ Nevertheless, concerns remain regarding the durability of repair especially in patients presenting concomitant injuries as meniscal or ligamentous lesions which are detectable in up to 80% of ACL ruptures.^10^

Unlike ACL reconstruction, which relies on graft integration independent of the native ligament's healing capacity, ACL repair depends on biological healing of the native tissue. Concomitant meniscal or ligamentous injuries may create an adverse inflammatory milieu that impairs this healing process, potentially elevating the risk of repair failure compared with reconstruction.^10^, ^11^, ^12^ Although some studies suggest that these concomitant injuries may compromise outcomes, others found no significant effect on patient‐reported results.^10^, ^11^, ^12^

As prior research focused primarily on short‐term to midterm results, there is a lack of data evaluating midterm outcomes of ACL repair with SA in larger patient cohorts.^7^, ^8^ The influence of concomitant meniscal tears and collateral ligament damages is still unclear in the midterm follow‐up.^11^ Some studies postulate that accompanying injuries may compromise stability and increase failure rates, whereas others report no significant impact on patient‐reported outcomes (PROs).^5^, ^12^

The purposes of this study were to evaluate midterm functional outcomes after ACL repair with SA and to determine whether concomitant injuries influence PROs. We hypothesized that patients with isolated ACL injuries would show superior functional outcomes compared with those presenting concomitant lesions.

## METHODS

The study was approved by the institutional ethics committee (number 2019‐00758), and all participants provided written informed consent at follow‐up in accordance with the Declaration of Helsinki.

This retrospective single‐center study was conducted at a specialized orthopaedic hospital. All consecutive patients undergoing arthroscopic ACL repair with SA between January 2017 and March 2019 were screened for eligibility.

### Patient Selection and Surgical Protocol

Eligible patients had magnetic resonance imaging (MRI)‐confirmed proximal ACL tears (Sherman I‐II) with preserved synovial sheath (Figure 1), surgery ≤14 days postinjury, and minimum 6‐year follow‐up. Excluded were midsubstance/distal tears, poor tissue quality, multiligament injuries requiring reconstruction, or prior ACL surgery. These patients underwent conventional ACL reconstruction.^4^, ^13^

**FIGURE 1:**
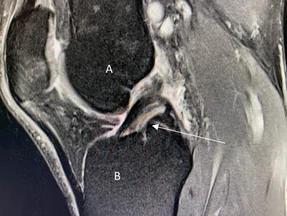
MRI investigation of the right knee joint with an ACL rupture in the proximal third (Sherman type I) and preservation of the synovial sheath in a sagittal section. (A) Femur, (B) tibia, and arrow, ACL. (ACL, anterior cruciate ligament; MRI, magnetic resonance imaging.)

The decision to perform ACL repair rather than reconstruction was based on intraoperative confirmation of proximal tear location, adequate tissue quality, and preserved synovial sheath as assessed by the operating surgeon (A.G.).

When feasible, surgery was performed on the day of injury (time from injury to surgery ≤24 hours). Treatment delays occurred because of external referrals, patient preference, or administrative requirements. Patients with treatment delays over 14 days were excluded from repair because of increased risk of knee joint stiffness and potentially reduced healing capacity.^14^ Associated concomitant injuries, for example, meniscal tears, were simultaneously addressed within the described procedure.

### Surgical Technique

A single senior fellowship‐trained orthopaedic surgeon (A.G.) performed all procedures to maintain technical consistency. The arthroscopic ACL repair technique was modified from the original one initially described by DiFelice et al.^4^ Intraoperative confirmation of proximal ACL tear was performed, and tissue quality was assessed based on integrity, fiber alignment, and preservation of the synovial sheath, as shown in Figure 2. A No. 2 FiberWire (Arthrex, Naples, FL) was passed through the ACL remnant approximately 1 cm distal to the tear site in a whipstitch configuration. The SA construct consisted of a 2‐mm FiberTape Internal Brace (Arthrex, Naples, FL) running parallel to the ACL from femoral to tibial fixation.

**FIGURE 2:**
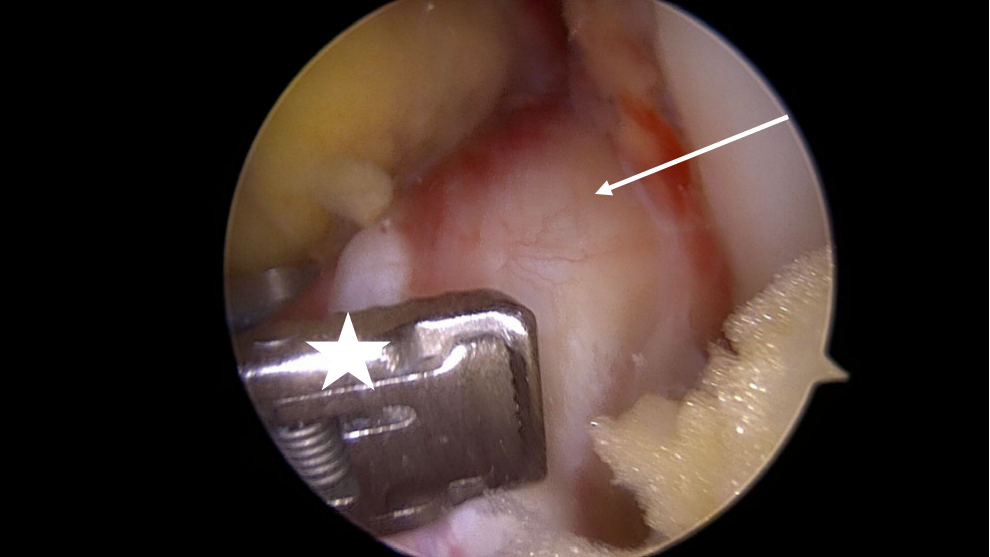
An intraoperative picture of the right knee joint, viewed from the anterolateral portal, during arthroscopy of the knee joint showing an arthroscopic suture passer (asterix, Fa. Arthrex) and a proximal ACL tear (arrow) with excellent tissue quality with an intact synovial sheath. (ACL, anterior cruciate ligament.)

The femoral tunnel was drilled inside out at the femoral footprint with the knee in full flexion, and a shuttle loop was advanced through this tunnel. The tibial tunnel was then drilled outside in using an aiming device at the anterior center of the tibial footprint, followed by passage of a second shuttle loop. Both shuttle loops and the FiberWire passed through the ACL remnant were retrieved via the anteromedial portal and shuttled through the tibial tunnel. The femoral shuttle loop was used to advance the SA construct together with the remnant suture through the tibial and femoral tunnels, and the ACL remnant was refixated to its anatomical origin at the femoral footprint (Figure 3). Proximal fixation was achieved by flipping the button at the femoral cortex, and distal fixation was performed with a 4.75 SwiveLock (Arthrex, Naples, FL) in full knee extension. To promote biological healing, additional microfracturing was performed at the femoral footprint. Collateral ligament injuries were diagnosed via preoperative MRI and intraoperative stress testing under anesthesia (valgus/varus stress test).

**FIGURE 3:**
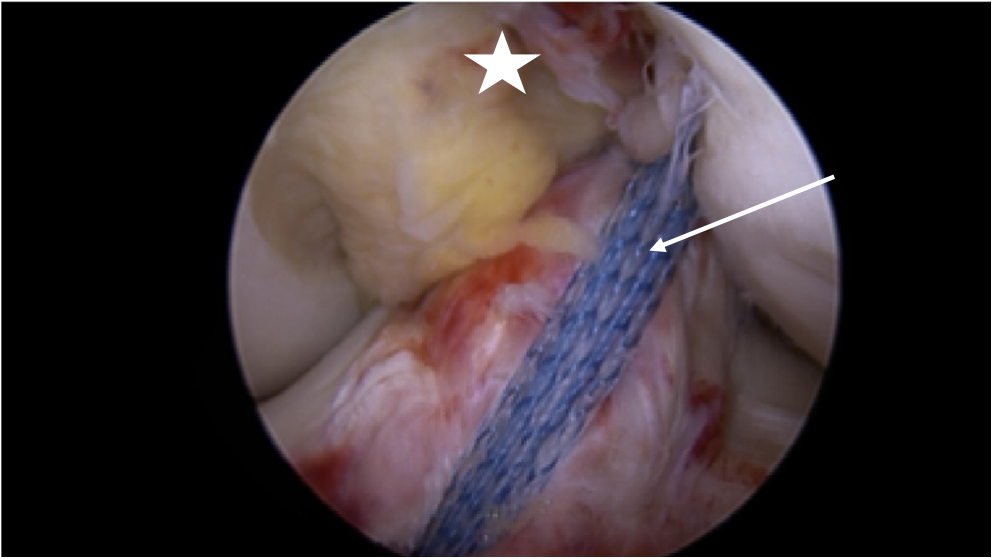
Intraoperative picture of the right knee joint from the anterolateral portal. Final pictures after performed ACL repair with SA (arrow) in a knee joint with a proximal ACL tear (Sherman type I) at the femoral insertion side (asterix) from the same patient as in Figure 2. (ACL, anterior cruciate ligament; SA, suture augmentation.)

Concomitant meniscal injuries were repaired when possible or partially resected if irreparable. Chondral lesions underwent debridement or microfracturing based on the characteristics of the defect.

### Rehabilitation Protocol

A standardized rehabilitation program was followed by all patients under supervision of experienced physiotherapists. Depending on the concomitant injuries, the standard protocol for ACL with SA included the following.

The mobilization was performed with partial weight‐bearing using crutches for the first 2 weeks followed by a progression to full weight‐bearing as tolerated in the next phase. If a meniscus repair took place, partial weight‐bearing was indicated for 6 weeks. Immediate postsurgery early range‐of‐motion exercises were initiated to minimize stiffness with the goal of achieving full extension within the first week and gradual flexion in the following.

Closed‐chain strengthening and neuromuscular training were introduced from week 4 onward. Jogging was typically permitted after 3 months with progression to sport‐specific movements after 6 months. Return to full stop‐and‐go sports was generally allowed at 9 to 12 months for patients showing adequate strength, stability, and functional performance. Individual return‐to‐sport data were not systematically captured in this retrospective study and could therefore not be reported as an outcome measure.

### Measures

Patient‐reported outcome measures (PROMs) were collected at a minimum follow‐up time of 72 months using 4 validated scaling systems via a questionnaire sent per email or mail: the Lysholm knee scoring scale (LS) (0‐100),^15^ International Knee Documentation Committee Subjective Knee Evaluation Form (IKDC) (0‐100),^16^ forgotten joint score (FJS) (0‐100),^17^ and Tegner activity scale (1‐10).^18^ Subgroup analyses were performed to assess the influence of (1) age, (2) sex, (3) time from injury to surgery (same day vs delayed), and (4) concomitant injuries (meniscal or ligamentous) on PROMs.

Demographic data (age, sex, and body mass index [BMI]), surgical data (date of surgery, time from injury to surgery, concomitant injuries, and their treatment), and revision information were extracted from electronic patient records.

At midterm follow‐up, no standardized clinical examination was performed because of the retrospective study design and extended follow‐up interval. Therefore, outcome assessment relied exclusively on validated PROMs. The cutoff age at 40 years was defined a priori based on previous literature and clinical experience, as patients above this age show reduced tissue quality and healing potential after ACL repair.^12^ As preoperative values were only available for the Tegner activity score (TS), minimal clinically important difference (MCID) analysis was performed exclusively for this outcome measure.

### Statistical Analysis

Statistical analysis was performed using Excel 2024 (Microsoft, Redmond, WA) and SPSS 31.0 (IBM, Armonk, NY). Data distribution was calculated using a test for normality (Kolmogorov‐Smirnov test). Data are presented as medians with interquartile ranges (IQRs 25%‐75%).

Paired comparisons (e.g., pre‐ vs postoperative Tegner) were analyzed using paired *t* tests or Wilcoxon signed‐rank tests as appropriate. Group comparisons (e.g., with vs without meniscal or collateral ligament injury, age ≥40 vs <40 years, same‐day vs delayed surgery) were initially performed using *t* tests or Mann‐Whitney *U* tests.

Clinically meaningful improvement was assessed by calculating the MCID using a distribution‐based method (½ standard deviation). A multivariate linear regression analysis was performed to evaluate the influence of meniscal injuries (medial and lateral), collateral ligament lesions (medial collateral ligament [MCL] and lateral collateral ligament [LCL]), age greater than 40 years, time from injury to surgery, BMI ≥ 25, and sex on postoperative PROMs. All predictors were entered simultaneously using the Enter method. All *P* values reported are two‐sided. Statistical significance was set at *P* < .05.

## RESULTS

Of the 176 consecutive patients presenting with acute proximal ACL rupture during the study period, 83 (47%) were not eligible for repair owing to intraoperative findings including poor tissue quality, insufficient remnant length, or midsubstance/distal tear patterns and subsequently underwent ACL reconstruction. The remaining 93 patients (53%) met the criteria for ACL repair with SA and form the study cohort. Those patients underwent arthroscopic ACL repair with SA between January 2017 and March 2019. Eighty patients (86%) were available for follow‐up. Thirteen patients (16.3%) underwent revision surgery after a median time of 35 months (IQR 8‐49). All revision surgeries consisted of ACL reconstruction. Revision surgery was performed because of a traumatic rerupture in 7 patients and in 6 patients because of chronic instability. These patients were excluded from further functional analysis.

Functional results were available for 67 patients (47 women, 20 men) with a median follow‐up of 79 months (IQR 75‐97, minimum 72 months) (Figure 4). On the day of surgery, median age was 42 years (IQR 33‐51) and median BMI 24 (IQR 22‐27).

**FIGURE 4:**
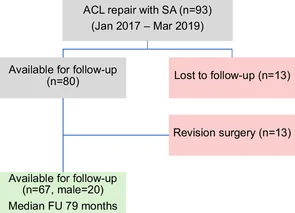
Study flow diagram illustrating patient inclusion, follow‐up, and analysis. A total of 93 patients underwent ACL repair with SA between January 2017 and March 2019. Of these, 13 were lost to follow‐up and 13 underwent revision surgery. The remaining 67 patients (20 men) were available for final follow‐up at a median of 79 months. (ACL, anterior cruciate ligament; FU, follow‐up; SA, suture augmentation.)

The median interval from trauma to surgery was 1 day (IQR 0‐1) with 81% of patients being treated within 24 hours after ACL rupture. However, 1 patient was included at 18 days per patient request for ACL repair.

### Functional Outcomes

A median LS of 92 (IQR 74‐100), an IKDC of 94 (IQR 71‐100), and an FJS of 86 (IQR 65‐94) were observed. As no preoperative values were available for IKDC, Lysholm, or FJS, cohort‐specific MCID calculation was not feasible for these outcomes. Median Tegner activity decreased from 7 (IQR 4‐10) preoperatively to 6 (IQR 4‐9) postoperatively (*P* = .03), a difference below the MCID, indicating no clinically relevant reduction in activity levels.

Cohort‐specific MCID for the TS was calculated using the ½ standard deviation method. As preoperative values were only available for this outcome measure, cohort‐specific MCID analysis was limited to the Tegner score. A total of 74.6% of patients maintained their activity level within the MCID threshold at final follow‐up.

### Concomitant Injuries

Concomitant pathology included 41 meniscal lesions (23 medial, 18 lateral) and 63 collateral ligament lesions (39 MCL, 24 LCL), whereas some patients had both MCL and LCL lesion. Among 18 lateral meniscus lesions, 10 underwent partial resection (56%), 4 were repaired (22%), 1 bucket‐handle tear was resected (7%), and 3 were repaired (15%). Of 23 medial meniscus lesions, 12 were resected (52%), and 3 were repaired (14%). Four bucket‐handle tears were resected (17%), and 4 were repaired (17%). All collateral ligament lesions were stable on intraoperative examination under anesthesia and required no additional surgical treatment. No significant differences in PROMs were observed between patients with or without concomitant meniscal or ligamentous injuries (all *P* > .05) (Table 1).

**TABLE 1:** Patient‐Reported Outcomes Stratified by Collateral Ligament Lesions

Scores	**MCL+ (n** = **39)**	**MCL− (n** = **28)**	***P*** **Value**	**LCL+ (n** = **24)**	**LCL− (n** = **43)**	***P*** **Value**
Lysholm score	95 (85‐100)	95 (90‐100)	.906	95 (80‐100)	95 (90‐100)	.983
Forgotten joint score	88 (74‐94)	84 (81‐94)	.437	81 (62‐94)	88 (81‐94)	.211
IKDC‐score	96 (89‐99)	96 (92‐98)	.939	97 (88‐99)	96 (91‐98)	.850
Tegner Score (preop)	7 (6‐7)	7 (6‐7)	.457	7 (6‐7)	7 (6‐7)	.488
Tegner score (postop)	6 (5‐7)	6 (6‐7)	.521	6 (6‐7)	6 (6‐7)	.576
Tegner score difference	0 (0‐0)	0 (0‐0)	.907	0 (0‐1)	0 (0‐0)	.201

*Note*: Functional outcomes comparing patients with and without MCL lesions (n = 39 vs n = 28) and LCL lesions (n = 24 vs n = 43). Values are presented as median (IQR 25%‐75%). No statistically significant differences were observed between groups for either MCL or LCL lesions.

IKDC, International Knee Documentation Committee; IQR, interquartile range; LCL, lateral collateral ligament; MCL, medial collateral ligament; postop, postoperative; preop, preoperative.

### Influence of Demographics on PROMs

Neither Lysholm, IKDC, nor Tegner score was significantly affected by the patients’ age (>40 vs ≤40 years). Also, the sex as well as the BMI did not significantly influence functional outcomes. The corresponding details are illustrated in Table 2.

**TABLE 2:** Patient‐Reported Outcomes Stratified by BMI and Sex

**Scores**	**BMI ≥ 25 (n** = **24)**	**BMI < 25 (n** = **43)**	***P*** **Value**	**Men (n** = **18)**	**Women (n** = **49)**	***P*** **Value**
Lysholm score	95 (85‐100)	95 (90‐100)	.704	95 (86‐100)	95 (90‐100)	.832
Forgotten joint score	81 (81‐89)	90 (81‐94)	.238	84 (80‐94)	88 (81‐94)	.902
IKDC‐score	96 (89‐99)	97 (91‐99)	.890	96 (89‐99)	97 (92‐99)	.336
Tegner score (preop)	6 (6‐7)	7 (6‐7)	.105	7 (6‐7)	7 (6‐7)	.064
Tegner score (postop)	6 (5‐7)	7 (6‐7)	.285	7 (6‐7)	6 (5‐7)	.061
Tegner score difference	0 (0‐0)	0 (0‐0)	.687	0 (0‐1)	0 (0‐0)	.704

*Note*: Functional outcomes comparing patients stratified by BMI (≥25 vs <25) and sex (men vs women). Values are presented as median (IQR 25%‐75%). No statistically significant differences were observed between groups across all assessed patient‐reported outcome measures.

BMI, body mass index; IKDC, International Knee Documentation Committee; IQR, interquartile range; postop, postoperative; preop, preoperative.

### Time‐of‐Surgery‐Related Factors Affecting PROMs

Univariate analysis showed no significant differences between groups. In the delayed group, the range of time to surgery was 2 to 14 days, with 1 protocol deviation at 18 days per patient request. The detailed results can be obtained from Table 3. Same‐day surgery was defined as surgery within 24 hours after injury.

**TABLE 3:** Patient‐Reported Outcomes Stratified by Time From Injury to Surgery (Same Day vs Delayed Surgery)

**Scores**	**Same Day (n** = **28)**	**Delayed (n** = **28)**	***P*** **Value**
Lysholm score	98 (90‐100)	95 (79‐100)	.562
Forgotten joint score	89 (81‐94)	81 (65‐94)	.610
IKDC‐score	97 (96‐99)	96 (89‐99)	.200
Tegner score (preop)	7 (6‐7)	7 (6‐7)	.973
Tegner score (postop)	6 (6‐7)	6 (6‐7)	.898
Tegner score difference	0 (0‐0)	0 (0‐0)	.914

*Note*: Comparison of functional outcomes between patients who underwent same‐day surgery (≤24 hours, n = 28) and those with delayed surgery (n = 39). Values are presented as median (IQR 25%‐75%). No statistically significant difference was observed across outcome measures. Same‐day surgery was defined as surgery within 24 hours after injury.

IKDC, International Knee Documentation Committee; IQR, interquartile range; postop, postoperative; preop, preoperative.

### Multivariate Regression Analysis

Multivariate linear regression analysis revealed no significant influence of concomitant meniscal or ligamentous injuries on any PROM. Neither medial nor lateral meniscus lesions were independently associated with Lysholm (*β* = −0.57, *P* = .823; *β* = +1.32, *P* = .630), IKDC (*β* = −0.48, *P* = .798; *β* = −0.59, *P* = .772), FJS (*β* = −5.73, *P* = .264; *β* = −0.83, *P* = .880), or postoperative Tegner scores (*β* = −0.17, *P* = .569; *β* = −0.26, *P* = .410). Similarly, MCL and LCL lesions showed no significant association with any outcome measure (all *P* > .05).

Among demographic variables, age greater than 40 years, BMI ≥ 25, and male sex showed no significant association with any outcome measure in the multivariate model. Male sex showed a nonsignificant trend toward higher postoperative Tegner activity levels (*β* = +0.57, *P* = .086).

Delayed surgery was identified as the only significant independent predictor across all models: a longer interval between injury and surgery was independently associated with lower postoperative TSs (*β* = −0.79 [95% CI − 1.49; −0.10], *P* = .025). No significant association with Lysholm (*β* = −0.67, *P* = .825), IKDC (*β* = −2.51, *P* = .268), or FJS scores (*β* = −7.22, *P* = .237) was observed. The overall model fit remained modest across all outcomes (*R* ^2^ = 0.039‐0.188; all *F* test *P* > .12). Details are presented in Table 4.

**TABLE 4:** Multivariate Linear Regression Analysis: Predictors of Patient‐Reported Outcome Measures

**Predictor**	**Lysholm Score** ***β*** **(95% CI)/*P* Value**	**IKDC Score** ***β*** **(95% CI)/*P* Value**	**Forgotten Joint Score** ***β*** **(95% CI)/*P* Value**	**Postop. Tegner Score** ***β*** **(95% CI)/*P* Value**
Medial meniscus lesion	*β* = −0.57	*β* = −0.48	*β* = −5.73	*β* = −0.17
	(−5.65; 4.51)	(−4.26; 3.29)	(−15.90; 4.44)	(−0.75; 0.41)
	*P* = .823	*P* = .798	*P* = .264	*P* = .569
Lateral meniscus lesion	*β* = +1.32	*β* = −0.59	*β* = −0.83	*β* = −0.26
	(−4.14; 6.78)	(−4.65; 3.47)	(−11.76; 10.10)	(−0.88; 0.37)
	*P* = .630	*P* = .772	*P* = .880	*P* = .410
MCL lesion	*β* = −1.90	*β* = −1.21	*β* = −3.02	*β* = −0.50
	(−7.31; 3.51)	(−5.23; 2.81)	(−13.85; 7.82)	(−1.12; 0.12)
	*P* = .485	*P* = .550	*P* = .579	*P* = .109
LCL lesion	*β* = +0.97	*β* = −0.96	*β* = −10.25	*β* = +0.37
	(−4.67; 6.60)	(−5.14; 3.23)	(−21.53; 1.04)	(−0.27; 1.02)
	*P* = .733	*P* = .650	*P* = .074	*P* = .254
Age >40 yr	*β* = +2.03	*β* = +0.98	*β* = +0.34	*β* = −0.18
	(−3.80; 7.86)	(−3.36; 5.31)	(−11.34; 12.01)	(−0.84; 0.49)
	*P* = .488	*P* = .653	*P* = .954	*P* = .596
Delayed‐surgery	*β* = −0.67	*β* = −2.51	*β* = −7.22	*β* = −0.79
	(−6.72; 5.37)	(−7.00; 1.98)	(−19.32; 4.88)	(−1.49; −0.10)
	*P* = .825	*P* = .268	*P* = .237	*P* = .025*
BMI ≥ 25	*β* = −2.32	*β* = −0.57	*β* = +1.51	*β* = +0.46
	(−7.62; 2.98)	(−4.51; 3.37)	(−9.11; 12.12)	(−0.14; 1.07)
	*P* = .385	*P* = .773	*P* = .777	*P* = .132
Male sex	*β* = +0.11	*β* = −3.03	*β* = −4.49	*β* = +0.57
	(−5.61; 5.84)	(−7.29; 1.22)	(−15.95; 6.97)	(−0.08; 1.23)
	*P* = 0.969	*P* = .159	*P* = .436	*P* = .086
n	67	67	67	67
*R* ^2^	0.039	0.085	0.125	0.188
adj. *R* ^2^	−0.094	−0.041	0.004	0.076
*F* test (p)	0.966	0.710	0.420	0.123

*Note*: Values represent unstandardized regression coefficients *β* with 95% CI and corresponding *P* values. Each model included the following predictors simultaneously: medial meniscus lesion, lateral meniscus lesion, MCL lesion, LCL lesion, age >40 years, same‐day surgery versus delayed surgery (same‐day surgery coded as 0, delayed surgery coded as (1), BMI ≥ 25, and male sex (n = 67). Sex variable coded women = 1, men = 0; *β* for male sex represents effect of male compared with female sex.

ACL, anterior cruciate ligament; BMI, body mass index; CI, confidence interval; IKDC, International Knee Documentation Committee; IQR, interquartile range; LCL, lateral collateral ligament; MCL, medial collateral ligament; postop, postoperative; yr, year.

* *P *< .05.

## DISCUSSION

The most important findings of this study were good to excellent PROMs, no significant impact of concomitant injuries, age, or sex on functional outcomes, but, in multivariate analysis, delayed surgery was the only independent predictor, associated with lower postoperative TSs and a revision rate of 16.3%. Conde et al. performed a meta‐analysis of 9 studies comprising 1049 patients, showing no significant differences in most PROs between ACL repair with internal brace and ACL reconstruction, with the exception of improved Knee injury and Osteoarthritis Outcome Score symptoms scores and stronger hamstring strength in the repair group.^2^

However, most included studies had follow‐up periods of only 24 months with repair group sizes ranging from 11 to 134 patients, and a significantly higher failure rate was reported in the repair group (risk ratio 3.62; 95% CI 1.16‐11.33). The present findings both support and extend these results, confirming comparable functional outcomes at midterm follow‐up in a cohort of 67 patients with a minimum follow‐up of 72 months. The revision rate of 16.3% is consistent with the elevated failure risk identified by Conde et al. Importantly, none of the studies in the meta‐analysis systematically evaluated the influence of concomitant injuries, a variable present in up to 80% of ACL ruptures, and multivariate regression identified delayed surgery as the only independent outcome predictor, providing clinically actionable information absent from existing meta‐analytic data. Together, these findings emphasize that modern augmentation techniques achieve comparable functional results to reconstruction while preserving native ligament biology.^19^

Vermeijden et al. further reported significantly higher FJS scores in repair versus reconstruction patients at 2‐ to 5‐year follow‐up (85.3 vs 74.3, *P* = .022), with particularly pronounced differences in patients older than 30 years, male patients, and those with BMI > 25.^20^ The present cohort achieved a median FJS of 86 at 79‐month follow‐up, consistent with the repair group in Vermeijden et al. The present results extend these findings by providing longer follow‐up (minimum 6 years vs 2‐5 years) and by showing, in contrast to Vermeijden et al., no significant age‐related differences in any PROM, which may reflect the additional mechanical protection of SA compensating for age‐related biological disadvantages.^20^

This study also adds evidence regarding concomitant injuries. In the present cohort, meniscal or ligamentous pathologies did not negatively affect midterm outcomes, questioning the traditional exclusion criteria proposed by DiFelice et al.^4^

This is consistent with short‐term data from Schneider et al., who found no significant impact of concomitant injuries on PROMs at 21 and 35 months in the same patient cohort at earlier timepoints,^7^, ^8^ and with Garside et al., who showed that meniscal repair combined with suture‐augmented ACL repair yielded comparable PROMs and reinjury rates to meniscal repair with ACL reconstruction at 2 years.^21^ The relatively low rate of meniscal repair in this cohort, with partial resection performed in most cases, did not appear to negatively influence PROMs, suggesting that meniscal management strategy may be less critical than previously assumed when ACL repair is performed with adequate SA.

It should be noted, however, that most collateral ligament lesions were stable on intraoperative examination and required no surgical treatment and that meniscal tears were predominantly managed with partial resection rather than repair. The conclusion that concomitant injuries did not impair outcomes therefore applies specifically to injuries of this severity and may not generalize to more severe pathology such as complete collateral ligament tears or complex meniscal lesions requiring extensive repair. Patient selection should therefore focus primarily on tear morphology and tissue quality rather than the mere presence of concomitant pathology.

Age has been considered a limiting factor for ACL repair with SA, with animal models suggesting reduced healing capacity in mature models and a systematic review by van Eck et al. recommending repair for younger patients.^22^, ^23^ In contrast, no significant age‐related differences were observed in the present cohort. Several factors may explain this: biological stimulation by microfracturing, the additional mechanical protection of the SA construct, preservation of native proprioceptive fibers, a standardized rehabilitation protocol, and careful patient selection focusing on Sherman type I to II tears with good tissue quality.^5^, ^19^, ^22^, ^24^, ^25^ These factors may compensate for age‐related disadvantages, supporting the concept of considering “biological” rather than chronological age and broadening treatment options for active older individuals.^26^

Additionally, older patients may have lower baseline activity levels, placing reduced biomechanical stress across the repair and potentially contributing to the absence of age‐related outcome differences, an effect not captured by chronological age alone. The subgroup of patients aged 40 years or younger was, however, relatively small (n = 18), and these findings should therefore be interpreted with caution.

Magarian et al. showed a significant difference in functional results when ACL repair was performed at 14 days versus 6 weeks postinjury, suggesting a negative effect of prolonged delay.^14^ Consistent with this, multivariate regression identified time from injury to surgery as an independent predictor of lower postoperative TSs. Although statistically significant, the clinical magnitude of this effect is modest and should be interpreted with caution given the overall low model fit. The absence of significant predictors for Lysholm, IKDC, and FJS scores may reflect the specialized infrastructure of the present institution, enabling same‐day MRI diagnostics and surgical decision‐making that allowed precise patient selection even in delayed presentations.

The definition of “delayed” in this cohort (typically <2 weeks) may represent a critical biological window where healing capacity is preserved, and the standardized SA technique may provide sufficient mechanical stability to mitigate the disadvantages of short delays.^14^, ^27^

More than one third of patients underwent same‐day surgery, which may optimize biological healing conditions but is not feasible in most clinical settings and represents a potential selection bias. In centers without immediate surgical capability, these results suggest that referral within the operative window may not compromise outcomes when using contemporary repair techniques.

The revision rate of 16.3% exceeds rates reported in comparable cohorts (3‐7%) and is consistent with the elevated failure risk identified by Conde et al. (risk ratio 3.62).^2^, ^28^, ^29^ Of the 13 revision cases, 7 were because of traumatic rerupture and 6 because of chronic instability, at a median time of 35 months (IQR 8‐49). Contributing factors may include the high preoperative activity level (median Tegner 7), inclusion of patients with concomitant lesions, single‐surgeon/single‐center design, and extended follow‐up of up to 97 months increasing the cumulative probability of detecting late failures. These findings underline the importance of thorough preoperative counseling regarding revision risk in high‐demand patients.

Although the present study evaluated mechanical SA, biological augmentation represents an actively investigated alternative. The bridge‐enhanced ACL repair procedure uses a bioactive scaffold to stimulate native ligament healing and has recently received postmarket approval. Shah et al. reported comparable PROMs to ACL reconstruction with an aggregate retear rate of 15% across 3 clinical trials and 0 retears in postmarket data, and a meta‐analysis confirmed comparable IKDC scores between Bridge‐enhanced ACL repair and bone–patellar tendon–bone reconstruction at 12 and 24 months.^30^, ^31^ Unlike SA, which provides mechanical stability, Bridge‐enhanced ACL repair directly stimulates biological healing, suggesting these approaches may be complementary rather than competing. Future prospective studies with control groups and standardized clinical assessments are needed to validate these findings.

### Limitations

In our investigation, several limitations need to be acknowledged when interpreting our results. The retrospective character of this study limits the ability to control potential disruptive factors and may lead to the possibility of a selection bias. The absence of a control group undergoing traditional ACL reconstruction leads to an exclusion of a direct comparison between surgical techniques. Functional assessment was based solely on PROMs without clinical examination at follow‐up. This may not fully capture subtle instabilities.

Furthermore, as no validated anchor question was available, patient acceptable symptom state and substantial clinical benefit analysis could not be performed. MCID assessment was therefore limited to the TS using a distribution‐based method. Individual return‐to‐sport data were not systematically captured at follow‐up, precluding analysis of time to return to activity, a key perceived advantage of ACL repair over reconstruction. Compliance with the rehabilitation protocol was not formally assessed, representing a limitation of the retrospective design.

In addition, 13 patients (16.3%) required revision surgery, a rate clearly exceeding that reported in comparable cohorts (3‐7%).^28^, ^29^ A further 13 patients (16.3%) were lost to follow‐up, introducing a relevant risk of survivorship bias and likely overestimating the true success rate.

Furthermore, the single‐surgeon and single‐center design limits generalizability. In particular, the infrastructure enabling same‐day MRI diagnostics and surgical intervention represents a setting not available in most clinical environments, and the excellent outcomes observed in same‐day surgery patients may therefore not be reproducible in standard practice.

## CONCLUSIONS

Arthroscopic ACL repair with SA achieved good to excellent midterm functional outcomes in a 6‐year follow‐up, with 74.6% of patients maintaining activity levels within the MCID threshold. However, the revision rate of 16.3% remains clinically relevant. Concomitant ligamentous and meniscal lesions of low severity did not result in inferior PROs in this cohort.

## DISCLOSURES

The author (J.C.T.) declares the following financial interests/personal relationships which may be considered as potential competing interests: J.C.T. receives grants or contracts from Stiftung Endoprothetik Deutschland, payment or honoraria for lectures, presentations, speakers bureaus, manuscript writing, or educational events from Implantcast Germany, Nuvasive Germany, Rimasys Germany, and support for attending meetings and/or travel from Implantcast Germany. The other authors (A.P.E., K.N.S., G.G., J.F.W., A.K., G.A.) declare that they have no known competing financial interests or personal relationships that could have appeared to influence the work reported in this article.

## FUNDING

Open Access funding enabled and organized by Projekt DEAL.
